# Quantitative Phenotyping-Based *In Vivo* Chemical Screening in a Zebrafish Model of Leukemia Stem Cell Xenotransplantation

**DOI:** 10.1371/journal.pone.0085439

**Published:** 2014-01-15

**Authors:** Beibei Zhang, Yasuhito Shimada, Junya Kuroyanagi, Noriko Umemoto, Yuhei Nishimura, Toshio Tanaka

**Affiliations:** 1 Department of Molecular and Cellular Pharmacology, Pharmacogenomics and Pharmacoinformatics, Mie University Graduate School of Medicine, Edobashi, Tsu, Mie, Japan; 2 Mie University Medical Zebrafish Research Center, Edobashi, Tsu, Mie, Japan; 3 Department of Bioinformatics, Mie University Life Science Research Center, Edobashi, Tsu, Mie, Japan; 4 Department of Omics Medicine, Mie University Industrial Technology Innovation, Edobashi, Tsu, Mie, Japan; 5 Department of Systems Pharmacology, Mie University Graduate School of Medicine, Edobashi, Tsu, Mie, Japan; University of Pécs Medical School, Hungary

## Abstract

Zebrafish-based chemical screening has recently emerged as a rapid and efficient method to identify important compounds that modulate specific biological processes and to test the therapeutic efficacy in disease models, including cancer. In leukemia, the ablation of leukemia stem cells (LSCs) is necessary to permanently eradicate the leukemia cell population. However, because of the very small number of LSCs in leukemia cell populations, their use in xenotransplantation studies (*in vivo*) and the difficulties in functionally and pathophysiologically replicating clinical conditions in cell culture experiments (*in vitro*), the progress of drug discovery for LSC inhibitors has been painfully slow. In this study, we developed a novel phenotype-based *in vivo* screening method using LSCs xenotransplanted into zebrafish. Aldehyde dehydrogenase-positive (ALDH+) cells were purified from chronic myelogenous leukemia K562 cells tagged with a fluorescent protein (Kusabira-orange) and then implanted in young zebrafish at 48 hours post-fertilization. Twenty-four hours after transplantation, the animals were treated with one of eight different therapeutic agents (imatinib, dasatinib, parthenolide, TDZD-8, arsenic trioxide, niclosamide, salinomycin, and thioridazine). Cancer cell proliferation, and cell migration were determined by high-content imaging. Of the eight compounds that were tested, all except imatinib and dasatinib selectively inhibited ALDH+ cell proliferation in zebrafish. In addition, these anti-LSC agents suppressed tumor cell migration in LSC-xenotransplants. Our approach offers a simple, rapid, and reliable *in vivo* screening system that facilitates the phenotype-driven discovery of drugs effective in suppressing LSCs.

## Introduction

Leukemia stem cells (LSCs) comprise a population of cancer stem cells (CSCs) in hematological malignancies. They possess characteristics similar to those of normal stem cells, specifically, the ability to serve as progenitor cells, but in this case they give rise to all cancer cell types, including chronic myelogenous leukemia (CML), rather than the cells of normal hematopoiesis [1]–[4]. LSCs represent a malignant reservoir of disease that is believed to drive relapse and resistance to chemotherapy [4]. Imatinib mesylate, a BCR-ABL tyrosine kinase inhibitor, has revolutionized the treatment of CML and as such is a model for targeted therapy in other cancers. However, in recent years, the efficacy of imatinib in disease eradication has been challenged [5] because of the resistance of LSCs [6], [7]. Moreover, resistance to the newer tyrosine kinase inhibitors, such as dasatinib and nilotinib, has also been documented [8], [9]. Therapeutic failure in the permanent eradication of leukemia by anti-cancer drugs such as imatinib has stimulated interest in LSC-targeted drug discovery as a rational cancer therapeutic strategy. Although the pathophysiological functions of LSCs cannot be demonstrated under culture conditions, compounds that inhibit their growth have recently been identified by *in vitro* screening [10]. Nonetheless, preclinical evaluation of their therapeutic potential is relatively slow mainly because of the very small population of LSCs available for testing in animal models [11]–[13].

Over the last few decades, a zebrafish-based screening method has emerged as a high-throughput and cost-effective alternative to other animal models and as such has been used to assess the efficacy and toxicity of several chemical compounds [14], [15]. Young zebrafish can be easily raised in 96-well plates and the maintenance cost is less than 1% of that of mice [16]. In addition, the transparent body wall of the fish enables phenotype-based screening of functional internal organs, which can be imaged using fluorescent and/or luminescent probes [17], [18]. As a cancer model, the immaturity of the young zebrafish immune system allows the xenotransplantation of human cancer cells into the fish as early as 48 h post-fertilization (hpf) [19]. The advantages of zebrafish xenotransplantation have been demonstrated in several studies in which *in vivo* fluorescent imaging was used to evaluate tumorigenesis, tumor angiogenesis, and metastatic phenotype [20]–[22]. However, despite the advantages of this method, image acquisition and quantification are labor-intensive and thus not conducive for high-throughput chemical screening. Here, we describe a rapid and phenotype-based zebrafish xenotransplant assay that is compatible with automated high-content imaging in 96-well plates. The method was tested by evaluating the efficacy of imatinib, dasatinib, parthenolide, TDZD-8, arsenic trioxide, niclosamide, salinomycin, and thioridazine in preventing LSC proliferation, tumor cell migration *in vivo*.

## Results

### Zebrafish xenotransplantation assay for screening LSC inhibitors

**Figure 1:**
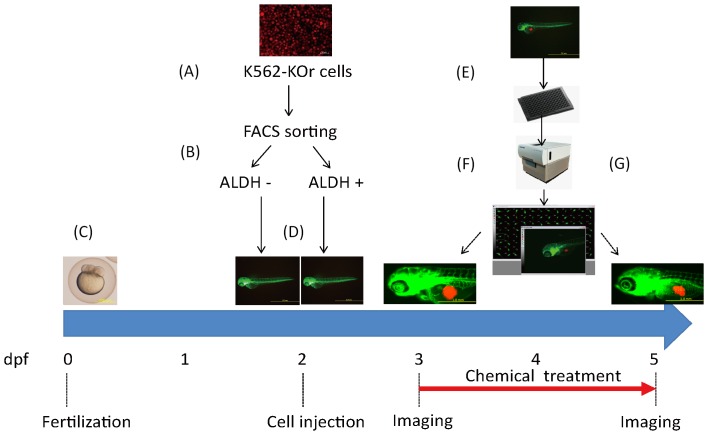
Schematic representation of the experimental design for LSC inhibitor screening in zebrafish. (A) Cultured K562 cells expressing a Kusabira-orange (KOr) fluorescent protein. Scale bar: 50 µm. (B) Sorting of ALDH- and ALDH+ cell populations. (C) A preparation of zebrafish embryos. Scale bar: 500 µm. (D) Xenotransplantation of ALDH- and ALDH+ cells into zebrafish at 48 hpf. Scale bar: 1.0 mm. (E, F) Xenografted zebrafish were transferred into 96-well plates at 72 hpf, imaged using a high-content imaging system, and then treated with the test compounds. Scale bar: 1.0 mm. (G) At 120 h (48 h after treatment), the xenografted zebrafish were imaged again to evaluate the effects of the chemicals. Scale bar: 1.0 mm.

A schematic representation of the experimental design is provided in Fig. 1. Cultured K562 (K562-KOr) cells stably expressing Kusabira-orange (KOr) fluorescent protein were subjected to fluorescence-activated cell sorting (FACS) to obtain aldehyde dehydrogenase-positive (ALDH+) cells and ALDH- cells, which were subsequently transplanted into the yolk sac of 48 hpf zebrafish. The xenotransplantation procedures are depicted in the Supporting Information (Fig. S1 and Movie S1). Twenty-four hours post-injection (hpi), cancer-positive fish with similar tumor mass were selected. The variation in Kusabira-orange integrated fluorescence intensity between the recipients at 72 hpf is shown in Fig. S2A. Xenotransplantation was successful in about 73% of the zebrafish. The average survival rate post transplantation at 72 hpf before treatment was above 84% throughout our study (Fig. S2B). The larvae were transferred to a 96-well plate and imaged under anesthesia using a high-content imager. The eight therapeutic test compounds were then added to the 96-well plate using an automated pipetting workstation. Forty-eight hours later, the larvae were imaged again and cancer progression, including tumor size and cell migration, was analyzed.

### ALDH+ K562-KOr cells have LSC properties

**Figure 2:**
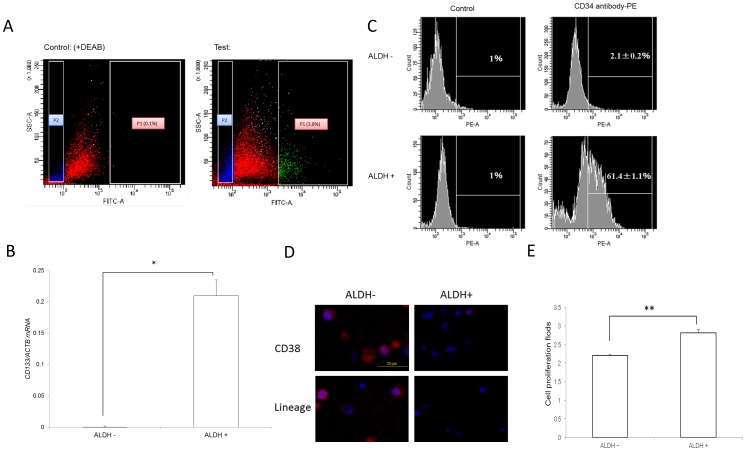
ALDH+ K562-KOr cells showed LSC properties. (A) FACS analysis for ALDH+ cells (P1 and P2 indicate the ALDH+ and ALDH- subpopulations, respectively). (B) qPCR for the CSC marker CD133 in ALDH+ cells (C) CD34-positive cells in the ALDH+ cell population (n = 3), *P<0.05. (D) The negative of CD38 and Lin in ALDH+ cells determined by immunofluorescent staining. Blue, nucleus; red, CD38 or Lin. Scale bar: 20 µm. (E) Cell proliferation at 72 h differed in ALDH- and ALDH+ cells (n = 3), **P<0.01.

**Figure 3:**
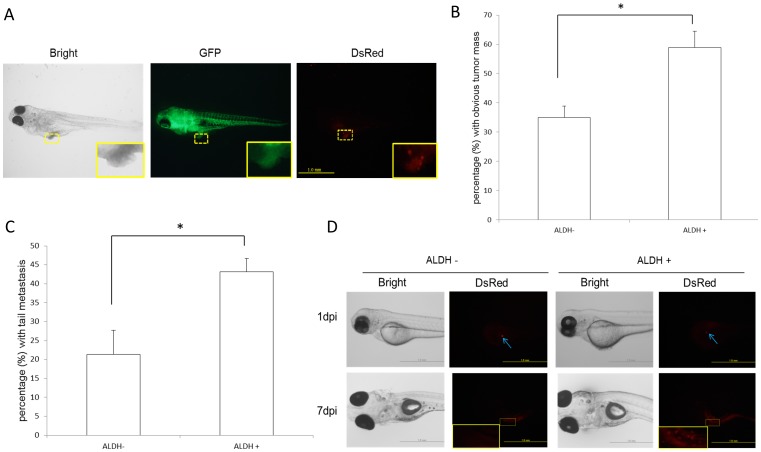
LSC ability in zebrafish xenograft. (A) Typical images of cancer xenografts at 6 days post-injection (dpi). Scale bar: 1.0 mm. The implanted tumor is framed in yellow (the magnification shows the outlined area). (B) In 6-dpi xenografts, ALDH+ cells exhibited greater tumorigenicity than ALDH- cells. ∗*P*<0.05, based on 3 independent experiments. (C) The distal (tail region) migration of ALDH+ cells was greater than that of ALDH- cells. ∗*P*<0.05, based on 3 independent experiments. (D) In single cell xenotransplants, ALDH+ cells proliferated at 7 dpi whereas ALDH- cells were no longer detectable. Scale bar: 1.0 mm. The implanted tumor is framed in yellow (the magnification shows the outlined area).

K562 cells with high ALDH activity (ALDH+) comprised ∼3.8% of the total cell population (Fig. 2A), similar to the yield in a previous study [23]. The LSC properties of these ALDH+ cells were determined by qPCR analysis of CD133 mRNA, a biomarker of CSCs [24]. CD133 expression was higher in ALDH+ than in ALDH- cells (*P*<0.01, Fig. 2B). FACS analyses of the two subpopulations for CD34, another CSC biomarker, showed that it was much more highly expressed in the ALDH+ (61.4%) than in the ALDH- (2.1%) population (Fig. 2C). CD38 and Lineage (Lin) were also negative in ALDH+ cells but not in ALDH– cells, as seen by immunofluorescent staining (Fig. 2D). In addition, the *in vitro* proliferation of ALDH+ cells was greater than that of ALDH– cells at 72 h (*P*<0.01, Fig. 2E). Six days after xenotransplantation (a relatively long duration for zebrafish), ALDH+ cells were more tumorigenic than ALDH- cells (Fig. 3A and B) and exhibited greater distal migration to the tail region (Fig. 3C).

**Figure 4:**
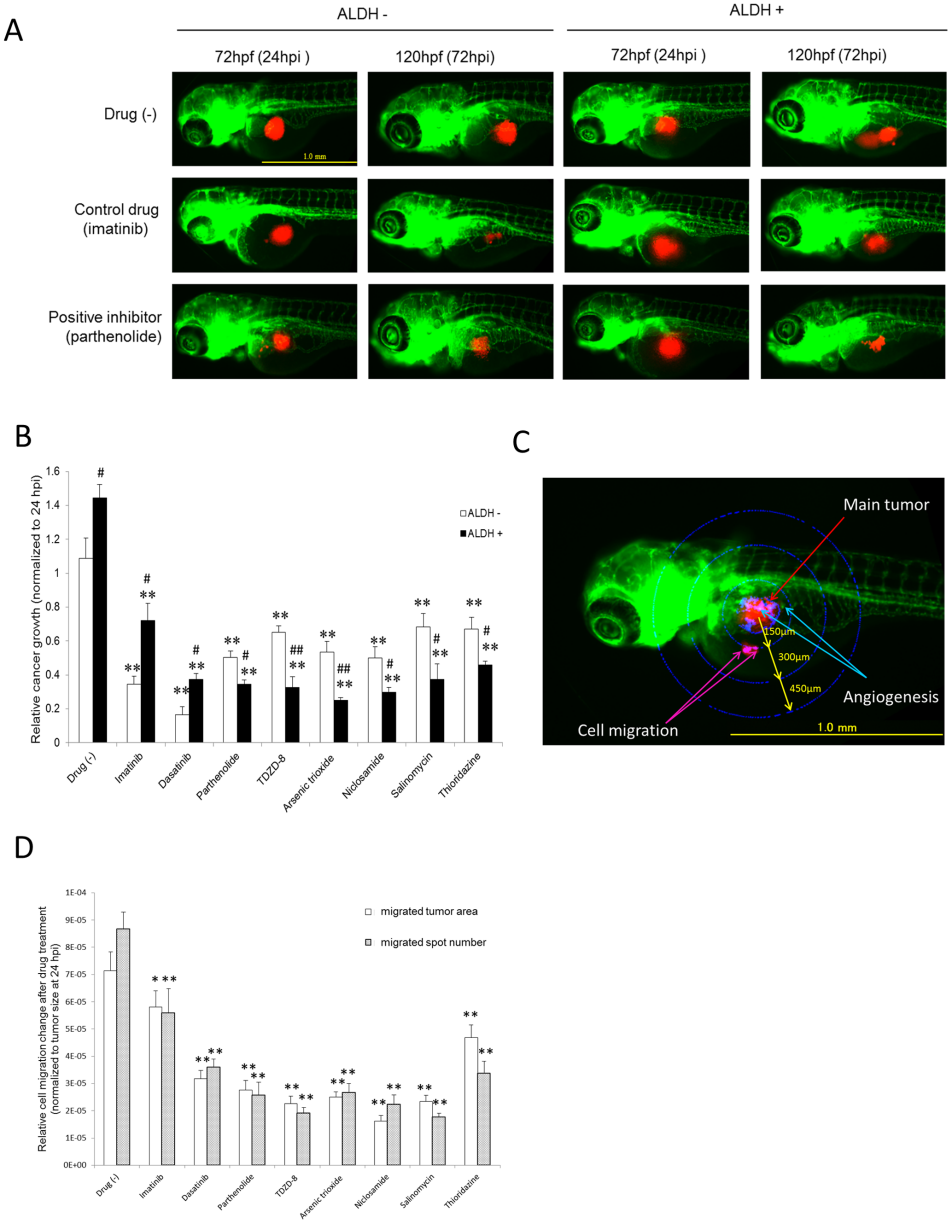
The effects of the test compounds on tumor inhibition in LSC-xenografted zebrafish. (A) Typical images of xenotransplanted zebrafish either not treated or treated with the control drug (imatinib) or an LSC inhibitor (parthenolide). Scale bar: 1.0 mm. (B) Tumor proliferation in xenotransplanted zebrafish. The test compounds (0.5 µM) were administered at 72–96 hpf. The ratio of the fluorescence intensity at 120 and 72 hpf was calculated as an indicator of the increase in tumor size, i.e. tumor proliferation (n = 15–25). ∗∗*P*<0.01 vs. no drug; #*P*<0.05 and ##*P*<0.01 vs. ALDH- cells. (C) Representative image of the main tumor, angiogenesis, and cell migration. Scale bar: 1.0 mm. (D) Quantitative analysis of cell migration. The number of migrated tumors and their sizes (area) were measured within concentric rings at a defined distance from the main tumor and grouped accordingly. The LSC inhibitors decreased the size and the number of migrated tumors in LSC xenotransplants (n = 15–25). ∗*P*<0.05 ∗∗ *P*<0.01 vs. no drug.

To validate these LSC properties in the zebrafish xenografts, we conducted an *in vivo* limiting dilution assay. Transplanted zebrafish with single cancer cell in transplant site were collected and the two cell populations (ALDH- and ALDH+) were analyzed. ALDH+ cells were observed to proliferate after 7 days while ALDH- cells were no longer detectable (Fig. 3D). Consistent with these findings, tumorigenesis capacity in zebrafish xenotransplanted with the ALDH+ population was also much higher than in fish xenotransplanted with ALDH- cells for 72 hpi (*P*<0.05, Fig. 4A and B). These results showed that the ALDH+ population of K562-KOr cells contained putative LSCs.

### Treatment of zebrafish xenotransplants with LSC inhibitors

The survival ratios of normal 3 days post-fertilization (dpf) zebrafish treated with the eight different therapeutic test compounds for 48 h are shown in Table 1. These data show that imatinib, dasatinib, TDZD-8, and arsenic trioxide had the fewest side effects, based on median lethal doses (LD_50_) >10 µM, followed by parthenolide and thioridazine (LD_50_: 1 µM–10 µM) and niclosamide and salinomycin (LD_50_: 0.5 µM∼1 µM). Because at a concentration of 0.5 µM none of the compounds caused obvious morphological changes, this concentration was chosen for use in the zebrafish xenografts. The efficacy of the chemicals in K562-xenotransplanted zebrafish was assessed based on the size of the main tumor as determined from its fluorescence intensity, which was measured using an automated imaging system. The size of the tumors formed by ALDH- and ALDH+ xenotransplants was significantly decreased by all of the tested chemicals compared to the non-treated control. Among them, imatinib was the weakest inhibitor of ALDH+ cells whereas dasatinib intensely inhibited both ALDH+ and ALDH- cells. However, ALDH+ cells were much more resistant than ALDH- cells to these two anti-cancer drugs. The other six CSC inhibitors (Table 2) were likewise screened for their selective inhibition of tumorigenesis in K562-KOr cells (Fig. 4A and B). In addition to the main tumor formed by the LCS xenografts, we evaluated tumor cell migration (foci separated further from the original mass) within concentric rings at a defined distance from the main tumor mass (Fig. 4C). Significant decreases in the size and number of migration spots (Fig. 4D) were obtained with all of the LSC inhibitors.

**Table 1:** Survival rate (%) of normal zebrafish treated with the test chemicals.

Chemicals	Concentrations(µM)						
	0.125	0.25	0.5	1	2.5	5	10
No drug	100±0	100±0	100±0	100±0	100±0	100±0	100±0
Imatinib	100±0	100±0	100±0	100±0	100±0	100±0	100±0
Dasatinib	100±0	100±0	100±0	100±0	100±0	100±0	100±0
Parthenolide	100±0	100±0	100±0	100±0	100±0	100±0	0±0
TDZD-8	100±0	100±0	100±0	100±0	100±0	100±0	100±0
Arsenic trioxide	100±0	100±0	100±0	100±0	100±0	100±0	100±0
Niclosamide	100±0	100±0	100±0	0±0	0±0	0±0	0±0
Salinomycin	100±0	100±0	100±0	0±0	0±0	0±0	0±0
Thioridazine	100±0	100±0	100±0	100±0	100±0	0±0	0±0

Zebrafish were exposed to different concentrations of the test compounds for 48 h from 72 hpf. Three independent experiments were performed (n = 10 under each condition per test).

**Table 2:** Characteristics of the test chemicals.

Compound	Leukemia type	Inhibited subpopulation	Main mechanism	Model	Reference
Imatinib	CML	Normal leukemia cells	Tyrosine kinase inhibitor	Mouse, human	[8], [9]
Dasatinib	CML	CD34+CD38-	Inhibits CrKL phosphorylation	Mouse, human	[8], [9]
Parthenolide	AML,CML	CD34+CD38-	Inhibits NF-κB, activates p53, stimulates ROS production	Mouse	[30]
TDZD-8	AML,CML,ALL	CD34+CD38-	Inhibits NF-κB, oxidative stress	Mouse	[31]
Arsenic trioxide	APL,CML	CD34+CD38-	Reduces PML, blocks NF-κB, stimulates ROS production	Mouse	[32], [35]
Niclosamide	AML	CD34+CD38-	Inactivates NF-κB, stimulates ROS production	Mouse	[36]
Salinomycin	CLL	CD44+	Inhibits the Wnt pathway and NF-κB, induces oxidative stress	Mouse	[37], [38]
Thioridazine	AML	CD45+CD33+	Inhibits DR signaling, antioxidant activity	Mouse	[39], [40]

CML, chronic myelogenous leukemia; AML, acute myelogenous leukemia; ALL, acute lymphoblastic leukemia; APL, acute promyelocytic leukemia; CLL, chronic lymphocytic leukemia; ROS, reactive oxygen species; PML, promyelocytic leukemia protein; DR, dopamine receptor.

**Figure 5:**
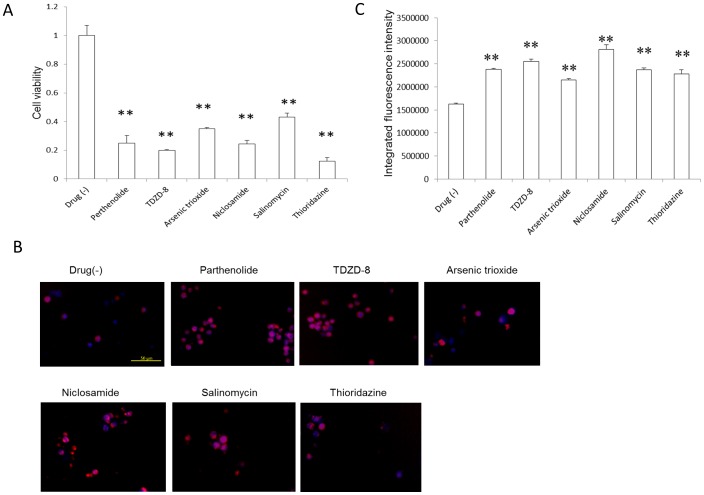
ROS overproduction by the LSC inhibitors. (A) LSC inhibitors (10 µM) significantly inhibited the *in vitro* proliferation of ALDH+ cells 24 h after treatment (n = 3), **P<0.01. (B) Typical images showing ROS production in ALDH+ cells. LSC inhibitors (10 µM) significantly induced *in vitro* ROS production in cells treated for 24 h. Blue, nucleus; red, ROS. Scale bar: 50 µm. (C) Quantitative analysis of ROS in the cells (n = 4), **P<0.01.

To explore the relationship of ROS and the anti-LSC effect, we cultured ALDH+ cells with 10 µM of the six LSC inhibitors except imatinib and dasatinib for 24 h. After treatment, the ALDH+ cell survival ratio decreased significantly (*P*<0.01, Fig. 5A). A determination of ROS status after chemical treatments showed that all six compounds induced the overproduction of ROS in ALDH+ K562 cells (*P*<0.01, Fig. 5B and C).

## Discussion

The advantages of therapeutically targeting the self-renewing LSC or CSC cell populations include less toxicity and fewer side effects. Moreover, this approach is more potent than standard chemotherapeutic agents, which non-discriminately target rapidly proliferating tumor cells but often fail to eliminate resistant cells. In this study, we developed a phenotype-driven *in vivo* screening method in which a zebrafish model is used to determine LSC inhibition.

In previous studies, cell populations with high ALDH activity were shown in serial or secondary transplantation assays to exhibit CSC properties [25], [26] and high ALDH activity has successfully been used to identify LSCs from clinical samples [27], [28]. The ALDH+ K562 cell population used in the present study expressed the putative CSC markers CD133 and CD34 and in zebrafish xenotransplants exhibited higher tumorigenesis and imatinib resistance than ALDH- cells, consistent with other reports [27], [29].

The LSC inhibition results support the findings of previous studies examining the efficacies and mechanisms of action of the tested compounds. The six that inhibited LSCs in our zebrafish model system are also potent inhibitors of CSCs, both *in vitro* and in rodent models [8], [11], [30]–[41]. With the exception of thioridazine, these compounds were reported to cause ROS overproduction and inactivate NF-κB [30]–[41]. In CSCs, low ROS levels protect cells from DNA damage during tumor seeding, suggesting that LSCs are more sensitive to oxidative stress than normal leukemia cells [42]. Thus, in tumor cells treated with ROS-stimulating compounds, a disturbance of the balance between ROS scavenging and production causes overwhelming ROS overproduction and in stem cell niches may trigger the differentiation of CSCs [43]. In the present study, ROS overproduction in ALDH+ K562 cells also indicated its importance in the anti-LSC effect. Consistent with the results of our cancer cell migration analyses (Fig 4D), in a study of prostate cancer salinomycin was shown to inhibit CSC migration by inducing oxidative stress, as demonstrated in a wound healing assay [37].

Cross-talk between ROS and NF-κB is well-established [44] and has been implicated in the mechanism of action of the NF-κB pathway inhibitor parthenolide, which preferentially inhibits the stem cell population of breast cancer cells [12]. The ability of thioridazine to block dopamine receptors may explain the reduced growth of CSC malignancies achieved with this drug [40], [45] and suggests a relationship between NF-κB and dopamine receptor signaling [46], [47].

A number of recent zebrafish leukemia xenograft studies indicated that zebrafish are a useful animal model in cancer research and chemotherapeutic drug screening [48]–[50]. Several methods have been developed for the *in vitro* evaluation of cell proliferation (MTT assay), metastasis (under-agarose migration assay), and angiogenesis (endothelial tube formation assay). Our zebrafish model provides an ideal platform for the simultaneous evaluation *in vivo* of LSC proliferation, angiogenesis, and metastasis (migration) as well as drug-related side effects. The small number of cells (100–200 cells/injection) required for the assay and the high-throughput screening (in 96-well-plate format) overcome the bottlenecks that arise because of the limited number of CSCs (∼0.1% of the total cancer cell population). In addition, with our zebrafish-based method multiple novel candidate anti-LSC agents can be tested in small amounts (nM or µM concentrations, 200 µl volume per animal). Thus, imaging-based LSC xenotransplant screening in zebrafish offers distinct advantages over other animal models and can greatly accelerate the phenotype-driven discovery of anti-LSC agents.

## Materials and Methods

### Ethical approval

All animal experiments were conducted according to the Animal Welfare and Management Act (Ministry of Environment of Japan) and complied with international guidelines. Ethical approval from the local Institutional Animal Care and Use Committee was not sought, since this law does not mandate the protection of fish. After the experiments, the fish were sacrificed at 5 (or 8/9) dpf by an overdose of anesthesia.

### Chemicals

All of the test compounds (imatinib, dasatinib, parthenolide, TDZD-8, arsenic trioxide, niclosamide, salinomycin, thioridazine) were purchased from Sigma-Aldrich (St. Louis, MO). Stock solutions (10 mM) were dissolved in dimethyl sulfoxide (DMSO; Sigma-Aldrich). For anesthesia, 100 ppm 2-phenoxyethanol (2-PE; Wako Pure Chemical Industries, Osaka, Japan) was diluted in E3 medium (5 mM NaCl, 0.17 mM KCl, 0.4 mM CaCl_2_, and 0.16 mM MgSO_4_).

### Zebrafish

The care and breeding of the zebrafish followed previously described protocols [51]. Because of the greater transparency of their bodies, which facilitates *in vivo* monitoring of tumor angiogenesis, nacre/rose/fli1:egfp zebrafish, obtained by cross-breeding nacre/rose mutants and fli1:egfp transgenic zebrafish, were used in the experiments [52]. Three days before xenotransplantation, individual female zebrafish were placed in mating tanks with males. The next morning, mating was initiated by light stimuli and the resulting fertilized eggs were collected. These eggs were incubated in E3 medium at 28°C, removing the dead eggs and replenishing the medium every day until the experiments were conducted.

### Preparation of K562-KOr cells

K562 cells were obtained from the RIKEN Cell Bank (Tokyo, Japan) and pre-cultured in RPMI1640 medium (Life Technologies, Carlsbad, CA) supplemented with 10% heat inactivated fetal bovine serum (Life Technologies), 100 U penicillin G/ml and 100 µg streptomycin (Sigma-Aldrich)/ml at 37°C in 5% CO_2_. The cells were transfected with the Kusabira-orange (KOr) fluorescent protein expression vector phKO1-MN1 (Amalgaam, Tokyo, Japan) using LipofectAMINE 2000 (Life Technologies) according to the manufacturer's instructions. Twenty-four hours after transfection, cells stably expressing KOr (K562-KOr cells) were selected in medium containing 800 µg geneticin/ml (Roche Diagnostics, Mannheim, Germany). After one week of culture, the KOr-expressing cells were purified by FACSAria flow cytometry (BD Biosciences, San Jose, CA) and further cultured.

### LSCs from K562-KOr cells

K562-KOr cells positive or negative for ALDH were sorted in an ALDEFLUOR assay (StemCell Technologies, Vancouver, Canada) followed by FACSAria flow cytometry (BD Biosciences), according to the manufacturer's instructions. To confirm the LSC character of the ALDH+ cells, CD34 expression was assayed by incubating the cells with anti-CD34-phycoerythrin (PE)-conjugated antibody (Beckman Coulter, Krefeld, Germany) followed by FACSAria flow cytometry according to the manufacturer's instructions. CD38 and Lineage (Lin) expression was confirmed using immunofluorescent staining with APC anti-human CD38 antibody (BioLegend, San Diego, CA, USA) and APC anti-human Lineage cocktail (BioLegend) according to the manufacturer's instructions.

### Total RNA extraction, cDNA synthesis, and qPCR

Total RNA was purified from the cells using the RNeasy mini kit (Qiagen, Hilden, Germany) according to the manufacturer's instructions. The first-strand cDNA was synthesized from 200 ng of total RNA using the SuperScript III cDNA synthesis kit (Life Technologies) with random primers (Life Technologies). RT-PCR was performed using Power SYBR Green Master Mix (Applied Biosystems, Foster City, CA) and a 7300 real-time PCR system (Applied Biosystems) as recommended by the manufacturer. The target gene was amplified using the primers CD133 (5′-ATC TGC AGT GGA TCG AGT TCT CT -3′ and 5′-ACA CAG AAA GAC ATC AAC AGC AGT AT-3′). The data were normalized with respect to the human housekeeping gene β-actin (ACTB), amplified with the primers 5′- TGT GCT ATC CCT GTA CGC CTC -3′ and 5′- GTA GAT GGG CAC AGT GTG GGT GA -3′.

### Cell proliferation assay

Sorted ALDH+ and ALDH- cells were cultured in 96-well plates at a density of 3000 cells per well in RPMI1640 medium supplemented with 1% heat inactivated fetal bovine serum, 100 U penicillin G/ml and 100 µg streptomycin ml at 37°C in 5% CO_2_. After treatment of the cells with the test compounds for 24 h, cell proliferation was measured using the CellTiter-Glo luminescent cell viability assay (Promega, Madison, WI, USA). Luminescence signals were measured in a Victor2 fluorescent plate reader (PerkinElmer Boston, MA, USA).

### Intracellular ROS quantification

The cells were fluorescently stained for intracellular ROS status by incubating them in 5 µM CellROX Deep Red detection reagent (Life Technologies) for 30 min, followed by three washes in PBS. After nuclear staining with 40 µg Hoechst 33342 dye (Dojin, Kumamoto, Japan)/ml for 5 min, images were captured to detect the intracellular ROS signal using the ImageXpress MICRO high content screening system (Molecular Devices, Sunnyvale, CA, USA). Cell fluorescence was quantified using the accompanying software.

### Leukemia cell xenotransplantation

Just before xenotransplantation, 48-hpf zebrafish were dechorionized using 2 mg pronase (Roche Diagnostics)/ml as described previously [53], anesthetized, and arrayed on a holding sheet. ALDH+ and ALDH- cells (1×10^6^ cells each) were separately suspended in 50 µl of Hanks' balanced salt solution (Life Technologies). The glass needles used to inject the cells were made from a GD-1 glass capillary (Narishige, Tokyo, Japan) using a PP-830 gravity puller (Narishige) and fine-polished with an EG-44 microforge (Narishige). The number of injected cells was counted microscopically by transferring the same volume of injected cells on glass slides using the same glass capillary tubes and injection pressure (FemtoJet, Eppendorf, Hamburg, Germany) in each experiment, as described in a previous study [54]. The avascular region of the yolk sac was then injected with a volume of the above-described suspension containing 100–200 cells using the glass needles and the FemtoJet injection system (Eppendorf, Hamburg, Germany). The xenotransplanted zebrafish were subsequently maintained at 32°C.

### High-content imaging

Twenty-four hours after xenotransplantation (72 hpf), the successfully xenotransplanted zebrafish were transferred in 50 µl of anesthetic solution into a 96-well imaging plate (353219; BD Biosciences). After gentle centrifugation (300 G, 30 s), the zebrafish were imaged live in an ImageXpressMICRO (Molecular Devices, Sunnyvale, CA) using the image acquisition program to automatically detect and image the fish in each well as follows: The overall well was imaged by prescanning, with 4-view (9-views for 48 wells) photographs obtained using a FITC filter (Semrock, Rochester, NY) and a 2-power lens (Plan Apo; Nikon, Tokyo, Japan). The zebrafish body was recognized based on the GFP intensity in the image and the stage was then moved such that the center of brightness was the center of view. Five images were taken continuously using a FITC filter and a 2-power lens, moving the system in the *z* direction by 40 µm each time. A composite image was then created from the best-focused images. For dual-wavelength (EGFP and KOr) imaging, serial radiography was performed using the same tetramethylrhodamine isothiocyanate (TRITC) method used for KOr and the best-focused composite image was created. A 4-power lens (S Fluor; Nikon) was used to obtain ten images, continuously moving the lens in the *z* direction by 20 µm each time. The Cool SNAP HQ (Roper Scientific, Tucson, AZ) CCD camera was used, with camera binning and gain both set to 1.

### Chemical treatment

After initial imaging of the fish, a JANUS automated workstation (Perkin Elmer, USA) was used to replace the anesthetic solution with 100 µl of the test compound in E3 medium. The 96-well plate was shaken for 30 s on an MTS2 shaker (IKA Labortechnik, Staufen, Germany) and then incubated at 32°C as described above. After 24 h, the medium was replaced with fresh chemical-containing medium, again using a JANUS automated workstation. Forty-eight hours after treatment (120 hpf), the zebrafish were imaged again as described above.

### Image analysis

Tumor size, and cell migration were analyzed using an imaging-based method and MetaXpress software (Molecular Devices). From the transplanted tumor clusters, the main tumor was identified using a multi-wavelength cell scoring application module based on the KOr (TRITC filter) images, calculating the area, total luminance value, and average radius of the tumors. Blood vessels were distinguished based on the detection of cell bodies, as described in the tumor angiogenesis image analysis program. Metastatic tumors were identified using the Transfluor application module. Concentric circles with radii of 150 µm, 300 µm, and 450 µm were drawn from the center of brightness and the number and size (area) of the metastases (migration) were calculated with respect to their distance from the center (0–150 µm, 150–300 µm, 300–450 µm, and >450 µm).

### Statistical analysis

Data are expressed as the mean ± SEM. Differences between two groups were compared using Student's *t*-test. For multiple comparisons, a one-way ANOVA followed by Dunnett's test for multiple comparisons was used. *P*<0.05 was considered statistically significant.

## Supporting Information

Figure S1 **Xenotransplantation procedures.**
(TIF)

Figure S2 **Description of xenotransplantation results.** (A) The average integrated fluorescence intensity (with respect to the volume of implanted cancer cells) of ALDH- and ALDH+ xenografts (24 hpi). There is no significant difference between the ALDH- and ALDH+ groups. NS, not significant. (B) Successful xenotransplantation and survival ratio.
(TIF)

Movie S1 **Xenotransplantation procedures.**
(WMV)
