# Supplementary figures and images for: Quantitative Phenotyping-Based In Vivo Chemical Screening in a Zebrafish Model of Leukemia Stem Cell Xenotransplantation
Source: PLoS One. 2014 Jan 15;9(1):e85439. doi: 10.1371/journal.pone.0085439 (PMC3893211; doi:10.1371/journal.pone.0085439)

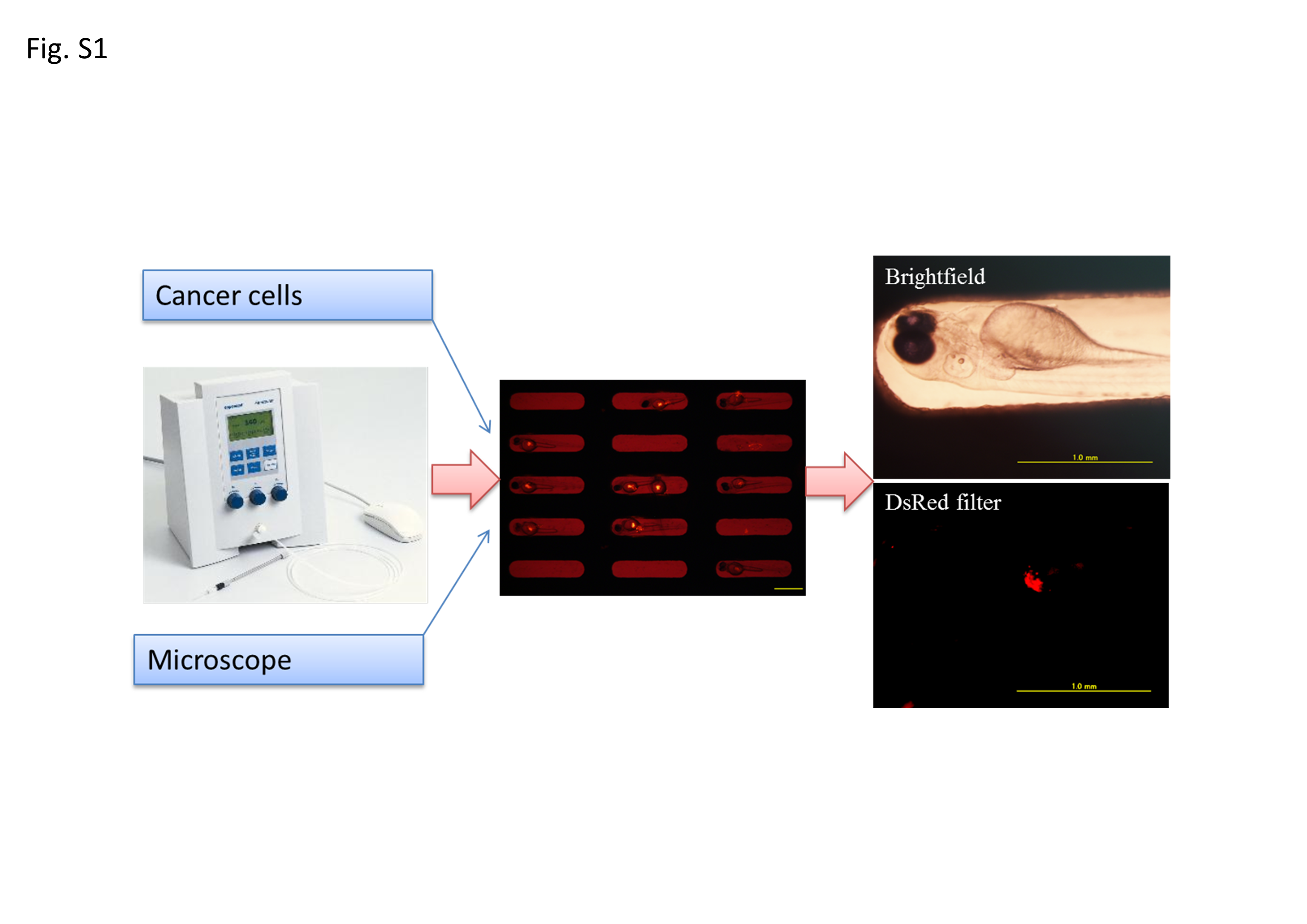

Supplement: Figure S1 — Xenotransplantation procedures. (TIF) [file pone.0085439.s001.tif]
